# N-Acetylcysteine and Other Sulfur-Donors as a Preventative and Adjunct Therapy for COVID-19

**DOI:** 10.1155/2022/4555490

**Published:** 2022-08-10

**Authors:** Heidi N du Preez, Colleen Aldous, Hendrik G Kruger, Lin Johnson

**Affiliations:** ^1^Catalysis and Peptide Research Unit, University of KwaZulu-Natal, Westville Campus, Durban, South Africa; ^2^College of Health Sciences, University of KwaZulu-Natal, Durban, South Africa; ^3^School of Life Sciences, University of KwaZulu-Natal, Durban, South Africa

## Abstract

The airway epithelial glycocalyx plays an important role in preventing severe acute respiratory syndrome coronavirus 2 entry into the epithelial cells, while the endothelial glycocalyx contributes to vascular permeability and tone, as well as modulating immune, inflammatory, and coagulation responses. With ample evidence in the scientific literature that coronavirus disease 2019 (COVID-19) is related to epithelial and endothelial dysfunction, preserving the glycocalyx should be the main focus of any COVID-19 treatment protocol. The most studied functional unit of the glycocalyx is the glycosaminoglycan heparan sulfate, where the degree and position of the sulfate groups determine the biological activity. N-acetylcysteine (NAC) and other sulfur donors contribute to the inorganic sulfate pool, the rate-limiting molecule in sulfation. NAC is not only a precursor to glutathione but also converts to hydrogen sulfide, inorganic sulfate, taurine, Coenzyme A, and albumin. By optimising inorganic sulfate availability, and therefore sulfation, it is proposed that COVID-19 can be prevented or at least most of the symptoms attenuated. A comprehensive COVID-19 treatment protocol is needed to preserve the glycocalyx in both the prevention and treatment of COVID-19. The use of NAC at a dosage of 600 mg bid for the prevention of COVID-19 is proposed, but a higher dosage of NAC (1200 mg bid) should be administered upon the first onset of symptoms. In the severe to critically ill, it is advised that IV NAC should be administered immediately upon hospital admission, and in the late stage of the disease, IV sodium thiosulfate should be considered. Doxycycline as a protease inhibitor will prevent shedding and further degradation of the glycocalyx.

## 1. Introduction

The pandemic surrounding coronavirus disease 2019 (COVID-19), caused by severe acute respiratory syndrome coronavirus 2 (SARS-CoV-2) infection, has been associated with immense economic and social disruption around the globe, apart from high mortality rates. More than two years later, there is still a need to find effective therapeutic solutions to prevent and treat established disease. Because of their various physiological functions in viral infection and inflammation, the use of N-acetylcysteine (NAC) and sodium thiosulfate (STS) [1, 2] was recommended in the very early stages of the pandemic, by the authors and various other scientists, for both prevention and treatment of COVID-19. It is important to understand the science underlying these recommendations, which will be explained in this review.

We have recently reviewed the pathogenesis of COVID-19 [3] that provides, inter alia, a comprehensive overview of sulfation of the glycocalyx (GL), as well as highlighting the importance of an intact sulfated GL for natural protection against SARS-CoV-2 infection [3, 4]. We provide a short summary here of our undersulfation hypothesis, so that the remainder of the manuscript (treatment of COVID-19) may be understood better. GL will refer to both the epithelial glycocalyx (EpGL) and endothelial glycocalyx (EnGL), unless specified.

**Figure 1:**
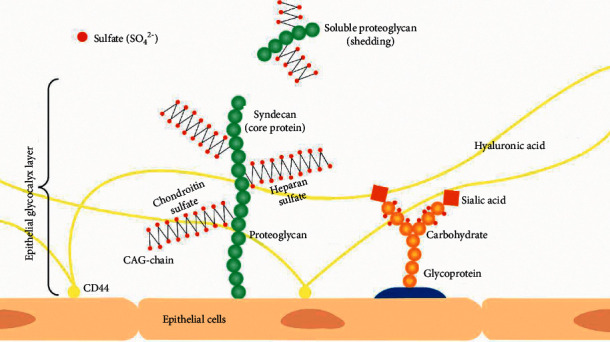
Schematic presentation of the epithelial glycocalyx (GL) in mammalian cells, forming a mesh-like structure that projects into the extracellular matrix (ECM). The proteoglycan presented is a syndecan with heparan sulfate and chondroitin sulfate glycosaminoglycan (GAG) side chains. The sulfate groups (SO_4_^2−^) are indicated in red dots, attached to the GAG and glycoprotein side chains. Hyaluronic acid is unsulfated and anchored to the receptor CD44 on the cell membrane. The glycoprotein consists of a protein base with carbohydrate chains (orange circles) extending into the extracellular space with sialic acid (orange square) bound at the terminal position of the oligosaccharide chains. Soluble proteoglycans and free GAG chains can appear in the extracellular space.

Since the GL surrounds all eukaryotic cells, the EpGL serves as the interface between our internal cells and the external world. It therefore serves as the first defence of the innate immune system, and if compromised, we are vulnerable to the onslaught of various pathogens, viruses, and environmental toxins [3]. The GL is a dense layer of proteins and carbohydrate chains that forms a mesh that extends into the extracellular matrix and is involved in many critical biological processes. Composed of membrane-bound glycoproteins, proteoglycans (PGs), and highly sulfated glycosaminoglycan (GAG) side-chains (Figure 1), the GL is particularly essential for barrier functions [5], as well as modulating immunity [3], inflammation [6, 7], and coagulation [8–11]. As a consequence of their interaction with multiple proteins, cell surface heparan sulfate (HS) PGs (HSPGs) are involved in developmental, regeneratory, infectious, and inflammatory processes [12]. It is important to note that the density and position of sulfate groups on the HS structure will mainly determine these HS-protein interactions [3, 13], and therefore the biological activity of sulfation.

Sulfation occurs in all tissues and involves the transfer of a sulfate group to various substrates, such as proteins, GAGs, lipids, hormones, various drugs, and toxins [14–18], [3, 19]. The availability of inorganic sulfate in the host was identified as the rate-limiting factor to sulfation [18, 20, 21]. The degree of sulfation and the trans-sulfuration pathway [22–24] is involved in many aspects of health, since it controls such a wide range of substances. The intact GL layer consists of many highly sulfated GAG chains, providing a negative charge density for the epithelial (Ep) and endothelial (En) surface layer [3, 25], and binding capacity for a variety of positively charged protein ligands [26]. The negative charge, mainly due to the presence of sulfate groups, is critical to biological and barrier function, especially in preventing the binding of negatively charged pathogens [3]. Changes in the charge density, and thus the degree of GAG sulfation, are often associated with various disorders [3, 27]. While different tissues and cell types vary in HS structure, the structure of HS can vary between individuals and with age. It seems evident that these differences in HS composition may contribute to tissue tropism and/or host susceptibility to infection by viruses and various other pathogens [3, 27–29].

Pre-COVID-19 vaccination, the elderly were most susceptible to SARS-CoV-2, where the typical profile of the critically ill patient was 65 years and older, presenting with comorbidities and ARDS [30, 31]. The main comorbidities related to COVID-19 were preexisting lung, heart and kidney disease, diabetes mellitus (DM) [32], obesity [33, 34], cancer, and cerebrovascular pathologies [35]. Dietary factors, such as low vitamin D and glutathione status, also increase susceptibility to COVID-19 [36, 37]. Today, more than two years later, immunosuppression in a younger population group seems to be a major predisposing factor for SARS-CoV-2 infection [38, 39]. Accumulating research relates dysfunction of the GL to ageing and these COVID-19 comorbid conditions [40, 41], which are also associated with chronic inflammation [3, 7, 34] and oxidative stress [42]. This highlights the probability that a disrupted GL is the main predisposing factor to COVID-19 susceptibility [3, 43]. As evident in COVID-19-related comorbid conditions, oxidative stress and chronic inflammation result in attenuation of the GL [7, 34, 42]. Moreover, En dysfunction increases systemic inflammation and oxidative stress, degradation of GL, and induces a procoagulant and antifibrinolytic state [35].

Since we are all exposed to SARS-CoV-2, the main question is why more than 80% of the world population did not become infected before the introduction of the COVID-19 vaccine, were asymptomatic, or showed only very mild symptoms [44–46], compared to the small percentage of people that became severely ill [22, 47]. The variable factor does not seem so much the expression of angiotensin converting enzyme 2 (ACE2) receptors, but rather the degree of GAG sulfation, which will affect the integrity of the GL, increasing susceptibility to SARS-CoV-2 infection and attenuating the ability to modulate inflammatory and coagulatory responses when the GL is undersulfated [3]. ACE2 expression is low in a well-preserved sulfated GL, which acts as a structural barrier [4]. Therefore, it is evident that the GL's structural and functional properties play a critical role in the pathogenesis of COVID-19. In this review, we will highlight the treatment strategies to best preserve the GL, with an emphasis on NAC and other sulfur donors as precursors to inorganic sulfate to support sulfation.

## 2. Glycocalyx

### 2.1. Structural Properties of the Glycocalyx (GL)

The extracellular matrix that surrounds all cells is a complex external layer of PGs, glycoproteins bound to sialic acid, GAGs, glycolipids, and proteins such as collagen [5, 48]. This glycoprotein polysaccharide covering is referred to as the GL. Plasma proteins such as antithrombin (AT) and albumin are also bound within the EnGL [49]. PGs are core proteins anchored to the apical membrane of Ep- and EnCs, with several GAG side-chains attached covalently to the PGs (Figure 1).

Glycosaminoglycan chains that bind to PGs are long linear, hydrophilic [50], negatively charged polysaccharides (PSs), namely, HS, chondroitin sulfate, dermatan sulfate, keratan sulfate, and nonsulfated hyaluronan [34, 48, 51, 52]. HS is the most studied and predominant GAG and acts as a receptor and reservoir for cytokines [53], enzymes, growth factor proteins, such as AT [12], and cell adhesion proteins, thereby modulating their distribution, concentration, and biological activity [54–56]. HSPG plays a critical role in maintaining and regulating various functions, including viral entry into target cells, vascular permeability [57], inflammatory and immune responses, and coagulation activity [3]. The GAG chain length and the number and location of sulfate groups in the various domains determine the binding affinity to the specific proteins [51, 58–60] and, therefore, biological function [12, 61, 62]. The degree of sulfation exerts the main influence on biological activity, therefore the GL's ability to modulate inflammation and coagulation, apart from barrier functions [3].

### 2.2. Sulfation

In mammalian cells, the sulfation process begins with the uptake of inorganic sulfate from the extracellular milieu [15], where the inorganic sulfate must be activated before reacting with the acceptor molecule. In mammals, this activated form of sulfate is sulfonucleotide 3-phosphoadenosine 5-phosphosulfate (PAPS), which is the universal sulfate donor for sulfotransferase (ST or SULT) reactions [11, 15]. STs transfer sulfate from PAPS to a specific position on the GAG structure [15], and therefore contribute significantly to the diversity of GAGs [3, 27].

### 2.3. Physiological Role of the Glycocalyx

Under normal healthy physiological conditions, HSPGs interact with and modulate the activity of several molecules [63], including protective enzymes such as superoxide dismutase (SOD) [64], cytokines [65], growth factors [25, 66], anticoagulant factors, albumin [57], Hedgehogs, Wingless, and protease inhibitors [67]. Therefore, apart from its essential role in barrier function and pathogen evasion [3], the GL will contribute to the regulation of coagulation, leukocyte adhesion [1, 65, 68], and protection against inflammation and oxidative stress [3, 49]. Any change in these functions is correlated with a wide range of pathophysiological consequences such as accelerated inflammation, capillary leak syndrome, consequent oedema formation [6], hypercoagulation, platelet hyperaggregation, and loss of vascular responsiveness [3, 25, 69]. The GL and HS do not only regulate physiological processes but are also implicated in many pathologies, including cancer, infectious and vascular diseases, and neurodegenerative disorders [1, 3, 5, 27, 56, 63, 70–73].

Of relevance to COVID-19, the primary role of the EpGL is the ability of a highly sulfated intact, negatively charged GL, to repel SARS-CoV-2 through electrostatic forces, thereby preventing cell invasion [3]. Most viral particles have a net negative charge at neutral pH, and SARS-CoV-2 is no exception. Various research reports confirmed the SARS-CoV-2 spike protein (SP) and net GRAVY score to be highly negative [74–77]. Nonetheless, if the GL is undersulfated and 3-O-sulfotransferase 3B (3OST-3B) is overexpressed, as is the case during chronic inflammatory conditions, the GL may facilitate SARS-CoV-2 entry [3]. Moreover, an undersulfated and degraded GL will not only increase susceptibility to SARS-CoV-2 infection, but will also hamper the ability to modulate the immune and inflammatory response and coagulation.

## 3. Sepsis Model, Degradation of the Glycocalyx

In COVID-19 patients that present with severe symptoms, sepsis has set in, which is a complicated condition that can be defined as severe organ dysfunction caused by an uncontrollable host response to infection [78]. After exposure to an infectious pathogen or inflammatory insult, alteration in the composition of the GL is one of the earliest features during sepsis [65, 68]. Following infection, acute injury, or inflammatory conditions, glucuronidases, such as heparanases (HPSEs) [58, 66, 79], reactive oxygen species (ROS) [65], and other proteases [5, 80, 81], cause shedding of HS and PGs [25, 57, 82]. Subsequently, adhesion molecules such as E-selectin and intercellular adhesion molecules are exposed on the denuded endothelium [6, 40, 80, 83], which result in accelerated inflammation [84], vascular permeability [58, 85], oedema, platelet aggregation, hypercoagulation, and a loss of vascular responsiveness [3, 40, 65, 68, 86, 87].

Pulmonary EnGL degradation increases the availability of En surface adhesion molecules to circulating microspheres. It contributes to neutrophil adhesion, leading to diffuse alveolar damage, interstitial lung oedema, and clot formation during acute lung injury in sepsis and ARDS [61, 68, 78]. There exists a clear association between alveolar epithelial and endothelial GL shedding and the establishment of ARDS [81, 82] and COVID-19 [4]. Moreover, circulating HS fragments are capable of influencing growth factors and other signaling pathways distant to the site of GL injury, which explains the systemic (i.e. para- or endocrine) consequences of GL degradation [51, 88], and supports the relationship between renal damage and the systemic proinflammatory state observed in sepsis [58] and COVID-19. Sepsis-associated induction of HPSE triggers degradation of vascular HS, leading to the collapse of the pulmonary and renal EnGL [51, 58]. While pulmonary EnGL loss contributes to lung injury via promotion of lung oedema and neutrophil adhesion, it was found that HPSE-mediated glomerular HS degradation could induce an early loss of glomerular filtration in the absence of kidney oedema or inflammation [51].

Apart from viral infections [82], multiple factors can cause degradation of the GL, including major surgery, ischemia/reperfusion, hypoxia/reoxygenation, mechanical ventilation [3, 48, 69], sodium overload [64, 80], ox-LDLs [5, 40, 86], sepsis, haemorrhagic shock, excessive shear stress, hypervolemia [48, 49], hyperglycaemia [68, 80], inflammatory cytokines such as tumour necrosis factor *α* (TNF*α*) [5, 85], interleukin (IL)-1*β*, IL-6, and IL-10 [48, 57, 58], matrix metalloproteinases (MMPs) [64, 65, 87], and reactive oxygen/nitrogen species [6].

Preserving the integrity of the GL is paramount both in the prevention and treatment of infectious diseases. Since most of the physiological properties of the GL are determined by the degree of GAG sulfation, ways to optimise inorganic sulfate, the rate-limiting substrate for sulfation, should be the primary focus of treatment. The intact Ep- and EnGL, with adequate sulfated GAGs, prevent viral infection and replication [3], determine vascular permeability, anticoagulant, anti-thrombotic, and anti-adhesive effects [11, 89], attenuate blood cell–vessel wall interactions [1], mediate shear stress sensing [40], enable balanced signaling, antioxidant properties [49], modulate inflammation [5], and fulfil a vasculoprotective role [25, 26, 48, 73]. In this review, we will expand on the role of NAC as a precursor to inorganic sulfate and its potential role as a preventive and therapeutic agent for combatting COVID-19.

## 4. NAC as a Physiological Precursor

**Figure 2:**
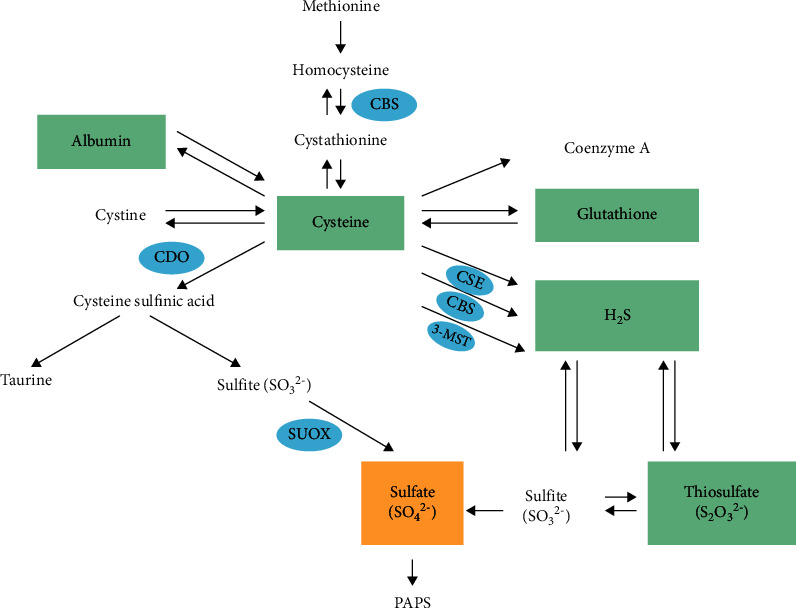
Sulfur metabolism diagram. The essential sulfur amino acid methionine converts to cysteine, a precursor to albumin, cystine, coenzyme A, glutathione, hydrogen sulfite (H_2_S), taurine, and sulfate (SO_4_^2−^). H_2_S can convert to thiosulfate (S_2_O_3_^2−^) and sulfite (SO_3_^2−^), which are oxidised to sulfate. The enzyme cystathionine-*β*-synthase (CBS) converts homocysteine to cystathionine, while cysteine dioxygenase (CDO) is responsible for the conversion of cysteine to cysteine sulfinic acid. Sulfite oxidase (SUOX) oxidises sulfite to sulfate. H_2_S is generated from cysteine by three different pathways through either CBS, cystathionine-*γ*-lyase (CSE), or 3-mercaptopyruvate sulfurtransferase (3-MST). Adapted with permission by CC by 4.0 [3].

Most of the literature to date focuses on NAC as a precursor to glutathione (GSH) [30, 42, 90–93]. However, it is important to realise that after free NAC enters a cell, it is rapidly hydrolysed to release cysteine (Cys), the rate-limiting substrate for intracellular GSH [94, 95], as well as hydrogen sulfide (H_2_S) [22, 30, 96, 97], inorganic sulfate [18], taurine [24, 98], Coenzyme A [99] and albumin synthesis [100] (Figure 2). NAC, therefore, has many physiological functions, apart from being an antioxidant precursor to GSH.

Since SARS-CoV-2 infection has been demonstrated to cause rapid depletion of sulfur amino acids (SAAs) due to oxidative stress or inflammation-induced proteolysis [22], amino acid replenishment becomes even more critical [18]. NAC treatment has been demonstrated to successfully replenish SAA levels, where a rapid increase in circulating Cys levels has been observed within hours following NAC supplementation [22]. During a rat study, the pharmacokinetics of NAC revealed that after oral administration, 77% NAC was maintained in the body and only 3% was excreted in faeces, while another human study indicated that after oral supplementation of NAC at 400 mg per single dose, the level of Cys increased to 4 mg/L in plasma [101].

### 4.1. Glutathione

Since Cys is the rate-limiting substrate in GSH and inorganic sulfate intracellular synthesis, low Cys levels will therefore result in lower GSH and inorganic sulfate levels. Through a series of redox reactions in the plasma, NAC directly affects the amino acid pool of extracellular cystine and intracellular Cys [18, 31]. NAC will favour GSH synthesis during oxidative stress. If there is a higher demand for GSH because of oxidative stress, such as during viral infection [102], extreme or endurance exercise [103], and hypoxic conditions [22, 94], it will have an inhibitory effect on inorganic sulfate synthesis. Lowered redox potential, common in high-risk COVID-19 patients, including older adults and those with uncontrolled DM, causes alterations in the TNF*α* receptor activity towards a proinflammatory state [22, 31, 95]. It is known that infection by RNA-viruses induces oxidative stress in host cells, and growing evidence indicates that viral replication is regulated by the redox state of the host cell [30, 94, 104, 105]. The aged patients with moderate and severe COVID-19 illness, and men had lower plasma levels of reduced GSH [30, 37], higher ROS levels, and greater redox status (ROS/GSH ratio) than COVID-19 patients with mild illness [37, 94, 106]. Erel et al. found that thiol levels decreased as the severity of the disease increased [107], while du Preez et al. hypothesised that low thiol levels/sulfur deficiency is an underlying cause of COVID-19 [3]. If these patients were low in GSH [108, 109], one can assume that Cys levels were low [102] and consequently inorganic sulfate, or it could be ascribed to a higher demand for Cys or inorganic sulfate during severe illness [106, 107, 110]. Bartonlini et al. demonstrated in vitro that SARS-CoV-2 infection impairs the metabolism of cellular GSH and its role in the redox homeostasis of cellular proteins, and results in changes in the composition of extracellular thiols [102, 111]. Even though it was found that COVID-19 patients with glucose-6-phosphate dehydrogenase (G6PD) deficiency [112, 113] and those with the glutathione S-transferase (GST) theta 1 (GSTT1)^−/−^ genotype or Ile 105Val glutathione S-transferase P1 (GSTP1) polymorphism, were more susceptible to infection and had higher mortality rates [114, 115], the availability of Cys will have a greater influence on redox status and outcome. The age-dependent decline of GSH and Cys in extracellular fluids has been hypothesised to not only be the actual causative factor [3], but also a molecular marker of increased risk of infection and development of serious COVID-19 [106, 107, 109, 116].

### 4.2. Hydrogen Sulfide (H_2_S)

Endogenous H_2_S supports basal, physiological, cellular bioenergetic functions, while the activity of this metabolic support decreases with physiological ageing [117]. Multiple biological regulatory roles for H_2_S as an endogenous gaseous transmitter have been established over the last decade [22, 97, 118]. H_2_S is produced, respectively, by cystathionine-*β*-synthase (CBS), CSE, and 3-mercaptopyruvate sulfurtransferase (3-MST) [22, 96, 118] (Figure 2). CBS and CSE serve as endogenous stimulators of cellular bioenergetics, while 3-MST serves as a regulator of cellular bioenergetics [118]. Moreover, during physiological oxygen reduction, H_2_S serves as a stimulator of electron transport in mammalian cells, by acting as a mitochondrial electron donor [117, 118]. It seems probable that stress and hypoxic states [97, 117] favour H_2_S as an “emergency” substrate that balances and complements the electron-donating effect of Krebs cycle-derived electron donors [119]. Of note is that the cell's free H_2_S concentration is likely to be constantly in dynamic equilibrium with a much larger pool of bound forms of sulfur, including thiol groups of proteins. Mitochondrial production of H_2_S starts with Cys [97, 106, 117], and the assumption can therefore be made that under hypoxic conditions, there would be an increased demand for H_2_S as electron donor [22], with a consequent inhibitory effect on inorganic sulfate and GSH synthesis, with Cys as the rate-limiting compound [119]. Even though one of the main H_2_S catabolic pathways in the mitochondria leads to the formation of thiosulfate, which is ultimately converted into sulfate [96], H_2_S would be favoured as an electron donor under hypoxic and stress conditions. This will affect the degree of HS sulfation [54]. Moreover, increases in intracellular H_2_S and ROS levels, mediated through hypoxic conditions [22], may synergistically induce membrane depolarisation [117]. This will result in increased levels of cytosolic calcium ions, which will lead to the activation of the endoplasmic reticulum (ER) stress response involved in the initiation of apoptosis [120]. Indeed, corona-derived viral protein deposits were found to result in ER stress and mitochondrial dysfunction in the affected EpCs [121].

Intrinsically synthesised H_2_S is extensively studied because of the role it plays as a proinflammatory mediator at high concentrations. Inhibition of endogenous H_2_S alleviates the diseased inflammatory condition, whereas exogenous H_2_S, released from natural sulfur compounds and SAAs, has shown protective effects in biological systems [117, 119]. H_2_S is stimulatory at lower concentrations, while it has an inhibitory effect on cellular bioenergetic functions at higher concentrations [118]. Inflammation, hypoxia, and calcium overload upregulate CSE gene expression and a simultaneous increase in H_2_S concentration [118, 119]. Conversely, H_2_S at low physiological concentrations [22, 97, 122] has been demonstrated to exhibit potent antiviral and anti-inflammatory properties [106, 123], mucolytic activity [96], as well as ameliorating various manifestations of inflammation, including ROS, nitric oxide, TNF*α*, and IL-6 [22, 122], and prevents endothelial dysfunction in cardiovascular-related pathologies [96]. Moreover, H_2_S attenuates pulmonary tissue injury by inducing ACE2 upregulation, while H_2_S may exhibit its antiviral activity against SARS-CoV-2 by interfering with both ACE2 and transmembrane protease serine 2 [22, 106]. COVID-19 patients with a more favourable outcome displayed higher circulating H_2_S levels than those found in patients with severe COVID-19 pneumonia [43, 96]. It seems evident that the H_2_S concentration must remain in a homeostatic balance to exert its protective effects. Even though Cys is the rate-limiting substrate for H_2_S production, pyridoxal 5′-phosphate (vitamin B6) is a very important cofactor, not only supporting CBS and CSE, but also the conversion of Cys to inorganic sulfate via CDO, along with iron. When supplementing with NAC to increase Cys levels, it is imperative to ensure a deficiency of the important cofactor nutrients does not impair the metabolic conversions in the sulfur metabolism pathway.

### 4.3. Inorganic Sulfate

Even though a small percentage of inorganic sulfate is found in foods and various sources of drinking water, the major source of inorganic sulfate for humans is from the biodegradation of dietary proteins. First, the essential sulfur amino acid methionine (Met) is converted to Cys [18]. Thereafter, the cytosolic enzyme, CDO, catalyses the conversion of Cys to cysteine sulfinic acid, the initial step in the transsulfuration pathway that leads to the formation of inorganic sulfate and taurine [124] (Figure 2). CDO is found primarily in the liver and brain, with some heart, kidney, and thyroid activity. It is known that the CDO-catalysed step is rate-limiting and that this metabolic route is responsible for the production of the majority of inorganic sulfate in vivo, since the absorption of inorganic sulfate across the gastrointestinal tract is relatively inefficient in humans [124]. The primary regulatory mechanism for CDO enzymatic activity functions at the posttranslational level. Under normal physiological conditions, high postprandial levels of Met or Cys will enhance the activity of CDO through inhibition of its degradation via the ubiquitin proteasome pathway. In the case of liver cirrhosis, however, CDO activity is significantly decreased despite high Met levels. It seems prudent that CDO expression is regulated at the mRNA level rather than the protein level. It was found that the proinflammatory cytokines, IL-1*β*, TNF*α*, and transforming growth factor-beta (TGF-*β*), downregulate CDO at mRNA level [98, 124]. This is evident in various health conditions with an autoimmune or inflammatory component, where high plasma levels of Cys with low plasma concentrations of inorganic sulfate are generally observed [124]. One can consequently expect reduced levels of HS sulfation during inflammatory conditions [3].

Most hospitalised COVID-19 patients in ICU are being placed on enteral nutrition [125]. Met is an essential amino acid, while Cys is conditionally essential since it is not always synthesised sufficiently, and preterm neonates have a relative inability to convert Met into Cys [126]. Most enteral and parenteral amino acid mixtures lack Cys since it is unstable in solution [126, 127]. A short-bowel syndrome rat model found that when enteral diets were supplemented with Cys and Met, improved gut mucosal and plasma cysteine/cystine redox potential and enhanced adaptive ileal mucosal growth were observed [126]. It is important to note that Met's contribution to the PAPS pool is 6–10 times lower than that of Cys [21]. Patients on enteral nutrition most likely do not get sufficient amounts of Cys [128], and during the cytokine storm seen in COVID-19, the conversion of Cys or NAC to inorganic sulfate appears to be reduced or inhibited due to the inhibitory effect of cytokines on CDO [124]. While adding Cys to enteral feed and intravenous (IV) NAC for the moderate to severely ill should be considered, IV STS should be explored in late-stage COVID-19, since it is more readily converted to inorganic sulfate. An easily digestible protein is recommended for mild to moderately ill patients, such as whey protein, high in Cys and albumin [129]. Another aspect to consider with enteral and parenteral nutrition is the very high calcium content in comparison to low levels of Cys and magnesium [126, 130, 131]. Both SARS-CoV and high levels of intracellular calcium ions would induce ER stress [120, 132]. Since high calcium levels also upregulate CSE gene expression [119], thereby increasing H_2_S levels and consequently membrane depolarisation [117], even more calcium ions will be released, creating a vicious cycle exacerbating inflammatory stress signaling [133] and apoptosis [120].

Cys is one of the amino acids in the hepatic precursor amino acid pool involved in both albumin and inorganic sulfate synthesis. The close arrangement between albumin synthesis and catabolic rates [134] suggests that a state of rapid equilibrium exists between the amino acid pools concerned with albumin and inorganic sulfate synthesis in the liver. It should be noted that whereas human serum albumin (HSA) is exclusively manufactured in the liver, hepatic inorganic sulfate also mixes with inorganic sulfate formed in other tissues in the extracellular fluid space. Inorganic sulfate synthesis in a healthy person mostly depends on the extent of reusing the sulfur from sulfate to synthesise new sulfur compounds. These conversions can occur through the intervention of microorganisms in the intestinal tract, although similar conversions can also occur in tissue cells [135]. Therefore, it seems probable that competitive inhibition exists in the synthesis between sulfate and albumin [134]. Pecora et al. indicated that the pathway of sulfate recruitment in the catabolism of SAAs was active in vivo [21]. They discovered that GAGs are undersulfated due to reduced extracellular sulfate uptake [134], caused by a mutation in diastrophic dysplasia sulfate transporter (Dtdst), which was reversed with the application of hypodermic NAC. This confirms that amino acid catabolism will contribute to the intracellular sulfate pool when extracellular sulfate availability is low. Therefore, the contribution of thiol compounds, such as NAC and HSA, to GAG sulfation, becomes significant by increasing the inorganic sulfate plasma concentration [21, 134]. Conversely, low levels of Cys or HSA will result in undersulfated GAGs. The dietary contribution to GAG sulfation has been reviewed extensively elsewhere [3].

Apart from NAC and HSA being sulfur donors, STS, methylsulfonylmethane (MSM) [136], and sulfated PSs [35, 137–140] will increase intracellular inorganic sulfate levels and thus exhibit antiviral and immune modulating properties. STS provides protection against ischemic brain injury and acute lung injury through inhibition of nuclear transcription factor kappa B (NF-*κ*B) activation and TNF*α*-induced production of cytokines and ROS, thereby preventing upregulation of IL-6. It is well established that STS is a potent antioxidant and anti-inflammatory agent, and it has been used to treat cyanide poisoning [141, 142] and calciphylaxis [143] with a remarkably safe track record. STS also acts as an H_2_S donor [122]. Taken together, these observations suggest a therapeutic potential for STS in COVID-19, taken orally, inhaled or intravenously [2, 122].

As a source of organic sulfur, MSM will increase the synthesis of GSH [144] and inorganic sulfate. Amirshahrokhi showed that MSM attenuates paraquat-induced pulmonary and hepatic injury in mice, demonstrated by the reduction of TNF*α*, malondialdehyde (MDA), and myeloperoxidase (MPO) levels, and an increase in SOD, GSH, and catalase (CAT) levels in lung and liver tissues [144]. Several studies demonstrated that MSM inhibits lipopolysaccharide-induced release of oxidative stress biomarkers, such as nitric oxide and prostaglandin E2 in macrophages, through downregulation of NF-*κ*B signaling [145, 146]. Kalman et al. reported that MSM potentially inhibits the translocation of the p65 subunit of NF-*κ*B to the nucleus, therefore minimising downstream events associated with local and systemic inflammation [147–149]. Indeed, supplementation with MSM will minimise the expression of many proinflammatory cytokines [144, 147, 148, 150]. This is confirmed in a study where MSM attenuated experimental colitis by reducing IL-1*β* levels and protected against hepatic liver injury by decreasing TNF*α* and IL-6 levels [144, 150]. In another study, MSM significantly mitigated lung and pancreatic histopathological changes, decreased serum amylase and MPO activity, and inhibited caerulein-induced IL-1*β* expression. Moreover, MSM reduced caerulein-induced H_2_S levels by decreasing CSE expression in the lungs and pancreas, and increased CD34+ expression [149]. Many of these beneficial properties of MSM could be attributed to increased intracellular levels of inorganic sulfate and therefore enhanced GAG sulfation.

### 4.4. Albumin

Under normal physiological conditions, oncotic pressure in the vascular system is maintained by HSA [40]. The shedding of HSPGs promotes albumin leakage and therefore reduces tissue turgor [134]. Hypoalbuminemia is frequently observed in patients with DM, hypertension, and chronic heart failure, and they are statistically most vulnerable to SARS-CoV-2 infection. Hypoalbuminemia is a known factor in sepsis, ARDS, and COVID-19 [59, 151]. It has been reported that low albumin levels are seen in almost 81% of COVID-19 deaths [151].

Hypoalbuminemia, vascular disease, and coagulopathy have all been linked to COVID-19 and have been shown to predict outcomes independent of age and morbidity [151]. All these conditions can be related to the degree of GAG sulfation [3]. HSA also plays a vital role in fat metabolism by binding fatty acids and maintaining them in a soluble form in the plasma. Hyperlipemia, therefore, occurs in clinical situations of hypoalbuminemia [152], which has been associated with COVID-19 [151]. Important research questions are: does hypoalbuminemia predispose patients to COVID-19, or is it a consequence of the disease—or both?

There is no doubt that the binding of SARS-CoV-2 to ACE2 receptors influences various processes, such as vasoconstriction, kidney injury, cardiovascular disease, apoptosis, and oxidative processes [153], but the effect of the degraded GL and shedding of HS is mostly overlooked in COVID-19 research. It has been well established that proteinuria occurs when the En barrier function is compromised [154], resulting in changes in plasma protein concentration, particularly HSA [151]. In a rat model, removal of HS by HPSE led to increased permeability for both albumin and ferritin, and injection of antibodies to HS led to acute selective proteinuria [155]. Studies showed that 59% of COVID-19 patients already presented with proteinuria upon hospital admission, where 22% of nonventilated patients and 90% of ventilated patients developed acute kidney injury [156], which confirms EnGL degradation in COVID-19 patients.

Plasma proteins, such as albumin [40, 57, 64, 68, 80] and fresh frozen plasma [48, 49], may protect the GL. After cold ischemia, it was observed that albumin supplementation significantly attenuated pronounced shedding of the GL [5], and consequently adhesion of leukocytes [25], with reduced interstitial oedema [49]. Meli showed that additional albumin in the perfusion medium positively affects the formation, support, and preservation of the EnGL [40]. Since albumin is degraded into amino acids [152], it will increase Cys levels and indirectly act as a precursor to inorganic sulfate. Moreover, albumin has immunomodulatory and anti-inflammatory [49], antioxidant, anticoagulant, and antiplatelet-aggregational properties [40].

NAC and Cys play an essential role as the rate-limiting substrate in many vital physiological processes, and it is crucial to understand the homeostatic balance between the sulfur substrates to maintain physiological function [43]. It seems evident that a low level of Cys or inorganic sulfate predisposes to COVID-19 [3], and paradoxically, there is a higher demand for these sulfur substances during severe illness [3, 18]. Moreover, since NAC, H_2_S, GSH, and albumin demonstrate many similar physiological functions, their donation of sulfur [43, 157] for inorganic sulfate synthesis, could probably be credited for their various physiological actions.

## 5. NAC as a Therapeutic Agent

Various research studies indicated that NAC modulates the immune system [158], inhibits viral binding and suppresses replication [123, 159], plus reduces inflammation [22, 30, 91, 94, 105, 160], apart from its antibiofilm [123] and antioxidant properties [22, 92, 161–163]. NAC also has clinical benefits such as in cough and dry eyes, and as mucolytic [94, 106, 164, 165]. Since NAC increases GSH and inorganic sulfate production, it can modulate various smoking-related end-points. It was shown that NAC attenuated several biomarker alterations, such as inflammation and lung airspace enlargement, in heavy smokers who received NAC (600 mg twice daily) for six months [30]. It was demonstrated that NAC increased GSH levels in plasma [102] and bronchoalveolar lavage fluid in humans [166], which should subsequently lead to an increase in inorganic sulfate levels. Various studies demonstrate the beneficial role of NAC in recovery facilitation after cerebral ischemia and traumatic brain injury and in the treatment of cerebrovascular vasospasm after subarachnoid haemorrhage [42]. NAC could therefore potentially be promising in ameliorating the long-term residual neuropsychiatric and neurocognitive conditions seen in long COVID [3, 153, 159, 167, 168].

Apart from being a precursor to GSH, various other mechanisms have been ascribed to NAC's antioxidant and anti-inflammatory properties. NAC downregulates inflammasome NLRP3 mRNA expression, therefore decreasing proinflammatory cytokine expression and release from activated mononuclear phagocytes; NAC inhibits the endotoxin-induced release of IL-1*β*, IL-8, and TNF*α*; prevents systemic endotoxemia and inflammatory response by improving gut barrier dysfunction [121], where previous studies have shown that COVID-19 has been associated with gut barrier dysfunction and systemic endotoxemia; and NAC downregulates programmed cell death protein 1 expression in CD4+ and CD8+ lymphocytes, therefore increasing their counts and longevity [158]. NAC also increases IL-2 production and proliferation, thereby promoting the activation and modulation of T and B lymphocytes [169, 170]. These immunostimulatory properties of NAC further justify its use for COVID-19, since reduced levels of CD4+ and CD8+ T lymphocytes have been observed in critically ill COVID-19 patients [171]. Its immunostimulant properties could also be beneficial to prevent subsequent opportunistic bacterial infections. Unlike glucocorticoids, NAC would seem to have a more favourable effect on modulating both the inflammatory and immune responses against infection [172, 173]. Many of these properties exhibited by NAC could likely be ascribed to increased intracellular inorganic sulfate levels.

Oxidative processes enhance viral infection by promoting replication in infected cells, inhibition of cell proliferation, and cell apoptosis induction [35, 95]. Studies found that NAC treatment significantly reduced the frequency of influenza in the elderly and the severity and duration of most symptoms [31, 95, 160, 162]. To replicate, RNA-viruses need active NF-*κ*B pathway support within host cells [158]. In a recent study, it was found that NF-*κ*B is a mediator of SARS-CoV-2 pulmonary pathology, since it triggers the production of numerous proinflammatory cytokines [162]. It was demonstrated that the suppression of hypoxia-inducible NF-*κ*B [92] significantly reduced the replication rate of mammalian coronaviruses. NAC has been shown to inhibit NF-*κ*B [30, 95, 105] and replication of influenza A viruses in human lung EpCs in a dose-dependent manner. NAC also reduced the production of proinflammatory cytokines, such as IL-6, IL-8, CXCL10, and CCL5 [106, 123, 165], hence reducing chemotactic migration of monocytes [160]. In addition, it has also been demonstrated that NAC inhibits replication of other viruses [165], such as HIV and respiratory syncytial virus [30, 160]. This means that NAC and other thiol donors should potentially inhibit SARS-CoV-2 as well [91], because of its ability to downregulate NF-*κ*B [35, 96, 160, 162]. The activity of monocyte chemotactic protein-1 (MCP-1) is upregulated by NF-kB, where MCP-1 amplifies inflammation and has been associated with the pathophysiological processes observed in COVID-19 patients [174]. Since NAC decreases both NF-*κ*B and MCP-1 levels [175], it demonstrates another mechanism for reducing inflammation in COVID-19 patients. Moreover, Debnath et al. showed that NAC bound to SARS-CoV-2 SP and resulted in a threefold weakening of SP binding affinity with ACE2 receptors [90]. Furthermore, an interesting interrelationship exists between vitamin D deficiency and sulfur metabolism [3, 113]. In animal studies, co-supplementation of vitamin D and NAC showed a greater benefit in increasing 25(OH) vitamin D levels, and in reducing oxidative stress and inflammatory biomarkers [36].

NAC was also shown to protect GL shedding, since HS is expected to restore the GL when it is shed from EnCs [68]. Pecora et al. indicated an increase in GAG sulfation due to NAC catabolism. Therefore, the contribution of thiols to sulfation becomes significant when their plasma concentration is increased [21]. An infusion of NAC attenuated hyperglycemia-induced degradation of the EnGL and coagulation [80], indicating the ability to restore the EnGL [43]. Many experiments with various sulfur-containing compounds confirmed the inhibiting effect of Cys on coagulation [3, 176]. De Flora et al. proposed the administration of NAC as a possible strategy to preserve En function and limit micro-thrombosis in severe forms of COVID-19 [30, 168]. NAC reduces thrombotic complications by inhibiting the activity of plasminogen activator inhibitor-1, which is a procoagulant positively correlated with severe cases of COVID-19 [177, 178]. Furthermore, due to NAC's ability to break disulfide bonds, it disrupts platelet aggregation and breaks the bond between blood cells and the clotting factor, thereby maintaining blood fluidity and oxygen flow in the specific area [106, 107]. NAC could therefore reduce the activation of the characteristic coagulation cascade of severe COVID-19 [3, 168]. Apart from the effect of sulfation in coagulopathy [3], NAC may also exert its anti-coagulopathy role by interrupting the vitamin K reducing electron transfer pathway, which otherwise could result in cerebral haemorrhage if co-administered with other anticoagulants and acetaminophen. Regular monitoring of the international normalised Ratio (INR) and prothrombin time (PT) is therefore recommended for patients taking anticoagulants and NAC simultaneously [179]. However, administration of NAC alone did not worsen haemorrhagic stroke outcome, suggesting that NAC exerts thrombolytic effects without significantly impairing normal hemostasis [168]. Daid et al. successfully treated a COVID-19 patient with intrahepatic haemorrhage with the application of IV NAC [180].

Various case reports using NAC to treat COVID-19 patients successfully have recently appeared in the literature [22, 30, 91, 123, 159, 160]. In a larger cohort study, it was found that IV NAC significantly improved disease conditions in 10 severely respirator-dependent COVID-19 patients, aged between 38 and 71 years, including one with G6PD deficiency. Apart from improved lung function, IV NAC administration significantly reduced inflammatory markers, such as C-reactive protein and ferritin [91, 94, 160]. NAC administration has also been successful as a prophylactic intervention for ventilator-associated pneumonia [31]. De Flora also describes various cases where IV NAC successfully treated ARDS and increased extracellular total antioxidant power and total thiol molecules [30]. Puyo et al. also described a successful case study where IV NAC and oral hydroxychloroquine (HCQ) were used in combination. Previous work has shown that HCQ and NAC modulate the innate immune system, reduce hypercoagulability, and inhibit thrombosis [181]. NAC also potentiates the vasodilator and antiaggregatory effects of nitric oxide, which is valuable in the context of acute heart failure, myocardial ischemia, and infarction [30], while it improved renal oxygenation in acute kidney injury in a rat model by decreasing free radicals [161]. Chavarría et al. compared the effect of the antioxidants vitamin E, vitamin C, NAC, and melatonin with pentoxifylline as adjuvant therapy in COVID-19 patients with moderate to severe pneumonia, where the simultaneous use of NAC (600 mg twice daily every 12 h) and pentoxifylline demonstrated the best effect in patients with severe symptoms [104].

Even though various clinical studies reported contradicting evidence [159], NAC could serve as a first-line drug for COVID-19 due to its structural and functional characteristics [3, 35, 106, 160, 162]. NAC is widely available, inexpensive, tolerable, and safe, and has been FDA approved for many years. It could be used in an “off-label” manner to improve therapeutic strategies for COVID-19 [37, 160]. When used for acetaminophen overdose, NAC is safe at doses of up to 980 mg/kg over 48 hours [181]. It is evident that NAC administered intravenously, orally, or inhaled, should suppress SARS-CoV-2 replication and may reduce symptoms if used timely. NAC's potential therapeutic benefits include scavenging ROS radicals extracellularly, replenishing intracellular GSH and inorganic sulfate, and suppressing cytokine storm and T cell protection, thus mitigating inflammation, coagulation, and tissue injury [30, 31, 160, 181, 182]. What is more, NAC inhibits the downstream activities post TNFɑ receptor activation and gene expression of TNFɑ and IL-6 while under oxidative stress [31, 182]. In a placebo-controlled study with peritoneal dialysis patients, administration of NAC (600 mg bid for eight weeks) has been shown to reduce the plasma levels of inflammatory markers, including complement C3 [30]. Furthermore, *in vivo* NAC modifies the function of the renin/angiotensin system, which is probably mediated by inhibition of ACE2 activity. By blocking ACE2, NAC will potentially protect patients from the deleterious effects of angiotensin-2, which seems to be a potentially useful strategy in SARS-CoV-2 infection [30, 123, 162]. NAC administration as first-line therapy, or as an adjunct therapy combined with other antiviral agents [30], may dramatically reduce hospital admission rates, the need for ventilation and mortality [35, 94, 160]. Altay et al. had great success with the application of a mixture of combined metabolic activators, such as NAC and nicotinamide adenine dinucleotide precursors, to facilitate a more rapid symptom-free recovery in COVID-19 patients [111].

Furthermore, since proteases, such as thrombin, have been reported to support the cleavage of syndecan 1 PG ectodomains, thus facilitating shedding of the GL, the therapeutic use of protease inhibitors should be considered [57]. Among the tested inhibitors is doxycycline [25, 57, 87], an appealing candidate as a repurposed drug in the treatment of COVID-19, with an established safety track record and solid preclinical rationale [183]. In several studies, researchers demonstrated that doxycycline inhibited matrix MMP activity, which significantly reduced GL shedding and, therefore, leukocyte adhesion to EnCs in response to inflammatory and ischemic stimuli [5, 25, 183]. Apart from NAC, the early administration of doxycycline will also play an essential role in preserving the GL. It is, therefore, apparent that a comprehensive integrative protocol is needed to treat COVID-19.

## 6. Treatment Recommendations for COVID-19

The main goal of any COVID-19 treatment regimen should be the preservation of an intact well-sulfated GL [3, 25, 49, 82]. Therefore, for the prevention and treatment of COVID-19, we propose the treatment options and dosages summarised in Table 1, which are based on clinical observation, the science [3] and evidence outlined in this review. Please note that the proposed treatments and dosages are based on what is currently cited in the literature and could only be adopted either under accepted hospital protocols or as part of clinical trials.

Even though we propose that NAC and STS can be used as first-line therapy, their application would complement any other treatment protocol as a safe adjunct intervention. COVID-19 is a complex disease that cannot be treated with a single drug approach. Various strategies to preserve the intact sulfated GL need to be applied. It is, however, essential to prescribe a sulfur donor such as NAC or STS when drugs that are administered need sulfation for their metabolism, such as steroids, aspirin, colchicine, acetaminophen, and nonsteroidal anti-inflammatory drugs [16, 186, 187]; [19, 165, 188, 189]. Apart from preventing the depletion of inorganic sulfate when these drugs are given, sulfur-donor supplements would complement the anti-inflammatory drugs through their synergistic immune-modulatory action [3]. Even though quercetin and melatonin have been shown to be very beneficial in the early treatment of COVID-19 [190–192], they also require sulfation to be metabolised. NAC should therefore also be considered as an adjunct to these early treatment protocols.

We propose that for the prevention of COVID-19 in the general population, especially in health care workers, frontline personnel, and those at high risk with comorbidities, a cysteine derivative such as NAC [30], carbocisteine, or erdosteine [106, 184] should be taken daily. These cysteine derivatives, and MSM as sulfur-donor [136], will favourably contribute to the Cys and inorganic sulfate pool. Rogliani et al. did a meta-analysis on the effect of NAC 1200 mg/day, carbocisteine 1500 mg/day, and erdosteine 600 mg/day on chronic obstructive pulmonary disease and found erdosteine to be superior in efficacy, compared to NAC and carbocisteine [184]. Even though NAC has been labelled as “low bioavailability” for decades, oral administration of NAC given within 8 to 10 hours of acetaminophen overdose displays the same detoxification capacity compared to the IV route. It was reported that 600 mg NAC in capsule form was able to reach a level of 16 *μ*M NAC in the peripheral blood within half an hour after administration [160]. Oral NAC at a dosage of 600 mg bid significantly decreased the frequency and severity of influenza [94, 95]. It is, therefore, expected that high dose oral NAC (1200 mg, bid) [94] can improve innate immunity through sulfation of the GL, and adaptive immunity by elevating GSH levels in lymphocytes, in addition to modulating neutrophil functions during the development of COVID-19 [3, 160]. As a preventive measure, oral NAC (600 mg bid) should be an effective and economical strategy to prevent SARS-CoV-2 infection and modulate the immune response [193]. Upon the first onset of symptoms, such as fever, sore throat, or dry cough, a higher dosage of oral NAC (1200 mg bid) should be considered to alleviate symptoms and accelerate recovery from viral infection [105, 160]. Note that high doses of oral NAC could result in intolerable gastrointestinal adverse effects such as nausea, vomiting, and diarrhoea [159]. Other micronutrients, such as zinc [194], selenium [106, 195, 196], magnesium, vitamins A, C, and D [113, 116, 197], should form part of a successful integrative protocol [110], while sulfur-donors such as MSM, allicin [157], or marine-derived sulfated PSs can also be considered. MSM is entirely safe and effective, taken at daily dosages of up to 4 g to prevent infection and modulate the immune response [136]. These self-treatment strategies to prevent viral infection with oral or inhalable NAC [22], or the use of other sulfur-donors, should help many SARS-CoV-2-infected patients to safely and cost-effectively recover at home. Since molybdenum, iron, and various B-vitamins are also important cofactor nutrients in sulfur metabolism, a balanced wholefood nutrient-dense diet should, therefore, form the cornerstone of any prevention and treatment regimen, supplying all macro- and micro-nutrients needed to prevent infection and maintain redox balance [3, 43].

In patients experiencing moderate to severe COVID-19 symptoms, IV NAC administration upon hospital admission should be considered as a standard practice of care, if allowed within the safety protocols of the hospital. The idea is to preserve and repair the GL to prevent a cytokine storm, and the sooner proper measures can be applied, the better the outcome that could be expected [22]. Shi & Puyo reported that patients with mild-to-moderate acute lung injury had significantly improved systemic oxygenation when intravenous (IV) NAC treatment (40 mg/kg/day) was given for three days, as well as reduced need for ventilatory support and lower mortality rate [160]. In another study, a 1200 mg/day oral dose of NAC was given for two weeks, which was sufficient to prevent deterioration due to severe respiratory failure requiring invasive or noninvasive mechanical ventilation in hospitalised patients with moderate or severe COVID-19 pneumonia, and reduced 14- and 28-day mortality [158]. Nonetheless, Taher et al. saw no clinical benefit with the application of NAC at a dose of 40 mg/kg/day diluted in 5% dextrose, given as a continuous intravenous infusion for 3 consecutive days, in patients with mild to moderate COVID-19-associated ARDS [159]. It should be noted that NAC was given at low dosage and in addition to standard-of-care treatment. A significant percentage of patients in both the NAC and control group received dexamethasone and other medications, which could reduce the degree of HS sulfation and therefore attenuated the GL [3, 198]. It is known that glucocorticoids may have intrinsic immunosuppressive drawbacks when applied at the wrong time, high dosages, and long term [3, 172, 173, 199, 200], while NAC is not immunosuppressive [159]. When higher concentration of IV NAC was given, better clinical outcomes could be expected. Shi & Puyo noted that high dosages of NAC will effectively reduce viral replication and significantly alleviate pneumocyte damage, as well as modulate immune responses and therefore prevent a cytokine storm [160]. NAC can, for example, be infused at a dose of 100 mg/kg for at least three days, which equals to about 1/3 of the total dose during a 3-bag regime. The aim should be to have at least an approximate NAC concentration in the blood of about 1 mM during infusion of the first bag to effectively treat virus-caused critical conditions, including pneumonia-mediated sepsis [159, 160]. However, Assimakopoulos et al. cautioned that very high doses of NAC may exert a prooxidant action, where it has been shown that the high doses may enhance Fe^2+^/H_2_O_2_-dependent oxidative stress and increase superoxide radical formation [158]. To offset potential ROS formation from high-dose NAC monotherapy, consider combining NAC with quercetin or vitamin C as powerful antioxidants to control free radical production [191, 201]. It seems evident that NAC as early intervention [121, 193] at the right concentration and enough exposure time is the key to an effective integrative treatment strategy. No difference has been observed between intermittent and continuous infusion of NAC regarding patient outcomes [160]. Nevertheless, when renal failure presents, an intermittent 3-bag regimen should be used. Although life-threatening anaphylactoid reactions were reported with IV administration of NAC, most of these reactions are mild in nature and easily manageable with a slower infusion rate [159]. Since HS can circulate for more than five days in the system as a result of a degraded GL, it is suggested that IV NAC should be given for at least 7 to 10 days, with doxycycline 100 mg once daily for 5 to 7 days as a protease inhibitor to attenuate shedding of the EnGL [25, 57, 87, 183]. Other antiviral medications can be added, but would be optional.

In the severe to critically ill COVID-19 patients, either IV NAC or IV STS should be considered, where the dosage and timeframe will be critical. STS is expected to be a better option to modulate the cytokine storm in the critically ill, since higher levels of cytokines will hamper the conversion of NAC to sulfate [98, 124], whereas STS will be more readily converted to inorganic sulfate (Figure 2). In a recent clinical trial, 135 severely ill late-stage COVID-19 patients received 300 mg/kg NAC over 20 hours or placebo [202]. The fact that there was no statistical difference between mortality rate and hospital stay for both groups indicates the importance of early initiation of NAC therapy [94] and considering STS application for late-stage disease. One should also consider the application of either NAC or STS at a lower dosage and for at least 7 to 10 days, since sulfated GAGs are not that easily restored [3]. A previous study demonstrated a relapse in inflammatory markers with early discontinuation of NAC treatment in COVID-19 [91]. In another study, IV NAC showed promising results in ARDS and acute lung injury patients at a loading dose of 150 mg/kg on the first day, followed by a dose of 50 mg/kg/day for three days. These patients not only showed improved oxygenation, but the mortality rate also dramatically decreased (35.7% vs. 76.9%) compared with control patients (*p* < 0.05) [160]. During another clinical trial, intravenous NAC (70 mg/kg body weight), given every 8 h for ten days, effectively decreased lung injury. It should be noted that NAC does not require dosage adjustments in renal or hepatic impairment [94].

We propose STS dosage to be the same as the standard treatment dosage given to patients with acute cyanide poisoning [185] or calciphylaxis [143, 203]. The recommendation is to give adults 100 mL (25 g) of STS (rate of 5 mL/minute) daily and when symptoms subside, administer every 2^nd^ or 3^rd^ day until there is no viral load present. In the paediatric group of 0–18 years, 1 mL/kg of body weight (250 mg/kg or approximately 30–40 mL/m^2^ of BSA) (rate of 2.5 to 5 mL/minute) should be given, not to exceed 50 mL of the total dose of STS [185].

The cytokine storm experienced in the critically ill will result in severe shedding of the GL that should be attenuated at all costs. One can consider the addition of dexamethasone with NAC as a combined anti-inflammatory therapy [22], as well as doxycycline at a dosage of 100 mg bid for 7 to 10 days.

### 6.1. Important Treatment Considerations

- Steroids, aspirin, colchicine, acetaminophen, and nonsteroidal anti-inflammatory drugs appear to enhance viral replication and aggravate Ep and En dysfunction and the cytokine storm [3, 19, 172, 173, 198, 199, 204] through the depletion of inorganic sulfate for its metabolism [16, 186, 187]; [19, 165, 188, 189, 205]. These medications should therefore not be prescribed without a sulfur donor such as IV NAC or STS.
- Whey protein, high in cysteine and albumin, should be added to the diet [18, 21, 129, 134] in mild conditions and add L-cysteine to enteral feed for severe to critically ill COVID-19 patients [126, 127].
- Administer albumin [40, 57, 64, 68] or fresh frozen plasma [48, 49] to restore the EnGL in the critically ill.
- Note that high dosages of vitamin C may cause hyperoxaluria through endogenous conversion of ascorbic acid to oxalate [125]. Both vitamin C [206] and oxalates [207] require conjugation through sulfation and contribute to Ep and En dysfunction if given at very high therapeutic dosages [3]. It appears best to give high-dose vitamin C together with IV NAC as a sulfur donor.
- Seen that NAC has powerful anticoagulatory effects, adjust dosages of blood thinning medications accordingly.
- Ventilation (VT) – avoid high tidal volume that could attenuate the lung EpGL [3, 87, 88, 208, 209]. However, low VT may not be the best approach for all patients with ARDS, since hypercapnia was common in patients with COVID-19-associated ARDS while using low tidal volume VT, which resulted in increased pulmonary dead space. Intermediate VT was used to correct hypercapnia efficiently in these patients while not excessively increasing driving pressure [210]. High-flow nasal oxygen remains the best option for severe COVID-19-related hypoxemic respiratory failure, particularly in settings outside the intensive care unit [211]. Excessive oxygen therapy can, however, be deleterious [112].
- Avoid hypervolemia and hypernatremia, which are injurious to the EnGL [65, 68, 69, 208]. Albumin is effective for volume repletion [68].
- Although Etanercept has been proposed for use in COVID-19 to reduce the mortality of toxic epidermal necrolysis (TEN) [212], Atef et al. proposed the use of 600 mg IV NAC every 8 h in surgical patients and ICU patients with TEN [213].
- Check for G6PD deficiency before HCQ is administered, and if present, give IV NAC to avoid the risk of haemolysis in G6PD-deficient patients [91, 181].
- Limit the use of antibiotics unless there is a known secondary infection. Gut dysbiosis seems to be an underlying risk factor for COVID-19 [3, 214].
- It is best to avoid the use of statins in COVID-19 patients, since they may cause rhabdomyolysis as a complication [112].
- Even though Ivermectin shows great potential as an inhibitor of SARS-CoV-2 replication [215], it is contraindicated when the blood-brain barrier (BBB) is compromised, as well as in the presence of multidrug resistance 1 (mdr-1) gene variants [216, 217] and deletions in glutathione S-transferases (GSTs) [218, 219], which can lead to increased drug accumulation in the brain, liver, and intestine [216], resulting in possible serious adverse events. Moreover, it is important to note that SARS-CoV-2 activation of cytokines, such as IL-1*β*, IL-16, and TNF*α*, cause injury to the BBB [153, 220] and decrease GSH levels [22]. Caution should therefore be taken with the use of ivermectin in late-stage COVID-19, and screening for polymorphisms in these genes should be considered.

Several clinical trials are currently evaluating the efficacy of NAC as therapeutic for COVID-19 [22, 94, 106, 123], but the various treatment considerations outlined in this review should be kept in mind with the application of NAC as therapeutic for a complex disease such as COVID-19. The timing and dosage of NAC supplementation would be the most critical factors determining the outcome, apart from the other measures needed to preserve the delicate GL layer. Even though well-designed clinical trials are needed to evaluate the effectiveness of a comprehensive NAC protocol, the use of NAC as an adjunct therapy should meanwhile be considered to ensure better clinical outcomes in the treatment of COVID-19 [106, 121, 161]. Enough evidence has been provided in the scientific literature for its safe and effective application [94]. Using a comprehensive treatment protocol with natural therapeutics, as opposed to a sole drug, would prevent the risk of highly pathological and resistant viral strains forming. Even though newly developed antiviral drugs, such as molnupiravir and paxlovid, show promising potential, they may result in more resistant viral mutations [221–223] and will not prevent GL shedding with all its pathological consequences.

After a comprehensive COVID-19 vaccine roll-out, the appearance of new variants with multiple mutations provides evidence that they are not effective; neither do they stop transmission nor infectivity, making it even more imperative to establish effective therapeutic alternatives to prevent viral infection. NAC would also appear to be a very good antidote against the possible adverse reactions experienced in susceptible individuals who received any of the COVID-19 vaccines. NAC may bind to the spike proteins, acting as a decoy to prevent binding to HS and consequent potential shedding of the GL. NAC will also facilitate detoxification of the adjuvants used in the vaccines, apart from restoring GSH levels depleted by the presence of ethylene glycol [3] and graphene oxide nanoparticles [94, 224–227].

## 7. Conclusion

Sulfated GAGs and various sulfur donor/thiol compounds regulate innate [3, 30] and adaptive immunity [123, 228] at various levels, as is well documented in the literature. What all sulfur donors have in common is the ability to supply sulfur for inorganic sulfate synthesis to ensure optimal GAG sulfation [3]. The degree and specific sulfation patterns of GAGs observed in a healthy GL attenuate the binding of pathogens, chemokines, and leukocytes to the EpC and EnC surfaces [156], thereby modulating immunity, inflammation and coagulation [3]. To maintain immune homeostasis, regulation of the initial inflammatory process in response to infection is crucial [22, 229]. Therefore, it is recommended that in moderate to severe COVID-19 illness, patients should be treated with IV NAC upon hospital admission. The safety of NAC was established with almost 60 years of experience in the prophylaxis and therapy of a variety of clinical conditions [123], even at very high doses and for long-term treatment. However, in the critically ill, IV STS should be considered, since the released cytokines might impede NAC oxidation into inorganic sulfate. As discussed in this review, the functions of the GL depend on an intact sulfated GL structure, with the degree of HS sulfation mainly determining biological activity [87, 230], with inorganic sulfate being the rate-limiting substrate to sulfation [3, 18, 20, 21].

Since NAC demonstrates multiple mechanisms of action, it is more likely to be effective than drugs having a single target. Moreover, since NAC addresses the underlying causes of COVID-19, it should have a beneficial effect on preventing and reducing long-COVID symptoms. Based on the herein discussed mechanistic premises, we propose using NAC in both the prevention and treatment of COVID-19. By better understanding the pathophysiological role of GAG sulfation [3], therapies can be better targeted at restoring the GL to ensure prevention and improved outcomes in COVID-19.

This review does not only emphasise the important clinical role of NAC in the prevention and treatment of COVID-19, but also highlights the physiological benefits of NAC as a precursor to inorganic sulfate. We believe that many of the physiological functions that have been attributed to NAC as a precursor to GSH in the literature to date, could in fact have been the action of inorganic sulfate, the rate-limiting precursor to sulfation. The application of NAC is therefore far-reaching beyond the treatment of COVID-19 or as an antidote to acetaminophen overdose.

## Figures and Tables

**Table 1:** Summary of a proposed therapeutic strategy to prevent and combat COVID-19.

Disease state	Sulfur-donor	Protease inhibitor	Comments
Prevention	Either NAC 1200 mg/day, carbocisteine 1500 mg/day, erdosteine 600 mg/day [184], or MSM 2 g/day [136].		Health care workers, frontline personnel, and those at high risk with comorbidities.

Mild disease	Double up the dosages indicated above.		Adequate dietary protein intake is important; they can add whey protein to the diet [21, 129, 134].

Moderate to severe symptoms	IV NAC upon hospital admission (100 mg/kg/day) for 7 to 10 days [160]	Doxycycline 100 mg qid 5 to 7 days [25, 57, 87]	Add L-cysteine to enteral feed [126, 127].

Severe to critically ill	Sodium thiosulfate—for 5 to 7 days and when symptoms subside, every 2^nd^ or 3^rd^ day. Adults: 100 mL (25 g) of STS (rate of 5 mL/minute). Paediatric 0–18 years: 1 mL/kg of body weight (250 mg/kg or approximately 30–40 mL/m^2^ of BSA) (rate of 2.5 to 5 mL/minute) not to exceed 50 mL total dose of STS [185] or IV NAC (150 mg/kg/day) for 7 to 10 days [160]	Doxycycline 100 mg bid 7 to 10 days [25, 57, 87]	STS might be a better option than NAC to modulate the cytokine storm in the critically ill. Add L-cysteine to enteral feed [126, 127]. Give albumin [40, 57] or fresh frozen plasma [48, 49]. Avoid high tidal volume ventilation [3, 48, 69, 123]. Avoid both hypervolemia and hypernatremia [65, 68, 69].

## Data Availability

All clinical recommendations made in the review article are based on published clinical trials, research, clinical experience and case reports, and are well referenced.

## Abbreviations

ACE2: Angiotensin converting enzyme 2
BBB: Blood-brain barrier
CDO: Cysteine dioxygenase
COVID-19: Coronavirus disease 2019
CSE: Cystathionine-*γ*-lyase
Cys: Cysteine
ER: Endoplasmic reticulum
En: Endothelial
EnGL: Endothelial glycocalyx
Ep: Epithelial
EpGL: Epithelial glycocalyx
GAG: Glycosaminoglycan
GL: Glycocalyx
GSH: Glutathione
G6PD: Glucose-6-phosphate dehydrogenase
HPSE: Heparanase
HS: Heparan sulfate
HSPG: Heparan sulfate proteoglycan
HCQ: Hydroxychloroquine
HSA: Human serum albumin
H_2_S: Hydrogen sulfide
IL: Interleukin
IV: Intravenous
Met: Methionine
MPO: Myeloperoxidase
MSM: Methylsulfonylmethane
NAC: N-acetylcysteine
NF-*κ*B: Nuclear transcription factor kappa B
PGs: Proteoglycans
ROS: Reactive oxygen species
SAA: Sulfur amino acid
SARS-CoV-2: Severe acute respiratory syndrome–related coronavirus 2
SOD: Superoxide dismutase
SP: Spike protein
STS: Sodium thiosulfate
PAPS: Sulfonucleotide 3-phosphoadenosine 5-phosphosulfate
ST or SULT: Sulfotransferase
TNF*α*: Tumour necrosis factor *α*.
VT: Ventilation
